# CS/Au/MWCNT nanohybrid as an efficient carrier for the sustained release of 5-FU and a study of its cytotoxicity on MCF-7†

**DOI:** 10.1039/d0ra08537e

**Published:** 2021-01-22

**Authors:** E. A. K. Nivethaa, S. Dhanavel, V. Narayanan, S. Narayana Kalkura, J. Sivasankari, N. Sivanandham, A. Stephen

**Affiliations:** Department of Physics, Anna University Guindy Chennai-25 India; Materials Chemistry & Metal Fuel Cycle Group, Indira Gandhi Centre for Atomic Research Kalpakkam India; Department of Inorganic Chemistry, University of Madras Guindy Campus Chennai 600 025 India; Department of Nuclear Physics, University of Madras Guindy Campus Chennai-25 India stephen_arum@hotmail.com stephen@unom.ac.in +044-22351269 +044-22202802; Crystal Growth Centre, Anna University Guindy Chennai-25 India

## Abstract

The chemical reduction method has been used to adeptly synthesize a CS/Au/MWCNT nanocomposite, to be used as a carrier for the effectual delivery of the anticancer drug 5-Fluorouracil. The work aims at utilizing the less investigated ternary nanocomposite system containing chitosan (CS), gold (Au) and MWCNT's to attain higher encapsulation efficiency and to enable a more sustained and prolonged release of 5-FU. This system has improved cytotoxicity when compared to the CS/Au binary system. The prepared sample has been characterized using various techniques that confirm the formation of the nanocomposite, encapsulation of 5-FU into the nanocomposite, the structure of 5-FU and Au in the nanocomposite and the formation of the polymer matrix nanocomposite. An increase in the encapsulation efficiency to 98% and loading efficiency to 43% is observed when compared to the binary composite, elucidating the importance of incorporation of carbon nanotubes into the nanocomposite. A reduction in the release percentage of 5-FU by 40% indicates a more prolonged release, which will enable a reduction of the number of dosages that need to be administered. This in turn leads to a reduction in the side effects posed by the drug 5-FU. Moreover, the effectiveness of the drug loaded nanocomposite system towards the inhibition of breast cancer cells, apparent from the attainment of 50% cell viability while taking sample concentrations as low as 25 μg ml^−1^, makes this ternary nanocomposite superior and significant.

## Abstract 2

The chemical reduction method has been used to adeptly synthesize a CS/Au/MWCNT nanocomposite, to be used as a carrier for the effectual delivery of the anticancer drug 5-Fluorouracil.

## Graphical abstract

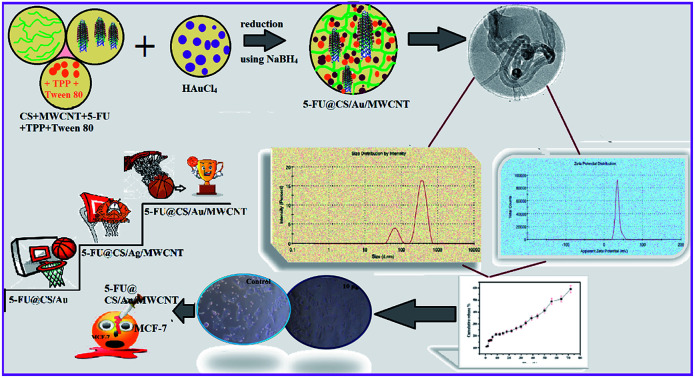

## 1 Introduction

Nanocomposites are multicomponent materials where mostly nanosized particles are incorporated into a matrix of standard material. Even though nanoparticles have been renowned for years due to their increased surface area and reactivity, which leads to increased bioavailability, nanocomposite systems are more advantageous as they retain the unique properties of all the individual components.^1^ Among the nanocomposite systems, polymer matrix nanocomposites have been widely used in biomedical applications. This is mainly due to the ability to engineer and amalgamate the properties of biopolymers like biodegradability, biocompatibility and non-toxicity with the properties of nanosized filler particles like the facilitation of communication with biomolecules on the cell surfaces and within the cells and also with receptors that are excessively expressed in case of diseases.^2,3^

Noble metal nanoparticles and nanotubes have been the most preferred nanofillers as they can be used as non-toxic carriers for drug and gene-delivery applications^4^ and due to their ability to enable the simultaneous loading of drug and targeting molecules,^5–7^ respectively. Moreover, the use of nanotubes leads to an increased drug loading efficiency and also enables the loading of desired molecules inside while, simultaneously imparting chemical features to the outer surface, allowing site-specific drug delivery.^5–7^ Carbon nanotubes have been reported to have the potential to deliver drugs directly to the targeted cells and tissues. Carbon nanotubes have been used for the delivery of anticancer drugs like paclitaxel and cisplatin.^8,9^ In addition, the ability of carbon nanotubes to act as carriers for a wide range of therapeutic molecules, their large surface area and possibility to manipulate their surfaces and physical dimensions for use in photothermal destruction of cancer cells have also been reported,^10–12^ due to which carbon nanotubes are chosen in the present study.

Amongst the noble metals, Au nanoparticles have gained importance as they provide a multifunctional platform to deliver drugs, to image and diagnose diseases and to preferentially administer electromagnetic radiation to disease sites (radiation is absorbed and converted to heat to destroy cancerous cells) which is called photo thermal therapy.^13–15^

The use of a combination of moieties mentioned above for the delivery of drugs has also been widely studied and reported. Reports on nanocomposites containing polymers,^16–18^ noble metals and metal oxides along with polymers,^19–23^ nanotubes and polymers,^24–28^ boron nitride and polymer^29^ and noble metal nanoparticles and metal oxides^30,31^ for the delivery of drugs are already available. Similarly carbon nanotube and noble metal containing nanocomposites^32,33^ as well as ternary composites containing biopolymer, noble metal nanoparticles and MWCNT^34^ have been used as drug delivery carriers. Among these, ternary nanocomposites exhibited better performance. Moreover, as there are only a very few reports on the use of ternary nanocomposites containing a noble metal along with MWCNT and biopolymer, we decided to investigate and throw spotlight on the use of CS/Au/MWCNT as a drug delivery carrier.

The present work thus, deals with the synthesis of CS/Au/MWCNT nanocomposites with and without drug encapsulation and their characterization. The prepared nanocomposites have been characterized using various techniques like XRD, FTIR, HRTEM, and UV-Vis. *In vitro* drug release studies have been performed using an UV-Vis spectrophotometer after the encapsulation of 5-FU into the composite systems. The cytotoxicity of 5-FU encapsulated nanocomposite towards MCF-7 cells has also been studied.

## 2 Materials and methods

### 2.1 Materials

Chitosan (CS) from Sigma Aldrich (low molecular weight and ∼85% deacetylated), Au chloride (HAuCl_4_) with ∼50% Au basis and 5-Fluorouracil with ≥99% purity from Sigma Aldrich, sodium tripolyphosphate (TPP) 98% pure from Alfa Aesar, Tween 80, ultra-pure from Alfa Aesar and sodium borohydride (NaBH_4_) extrapure 99% purity from Finar reagents were used for synthesis. Dimethyl sulfoxide (DMSO) with ≥99% purity was purchased from Sigma Aldrich. All chemicals used were of analytical grade. All experiments were carried out using double distilled water.

### 2.2 Preparation of CS/Au/MWCNT and 5-Fluorouracil encapsulates CS/Au/MWCNT (5-FU@CS/Au/MWCNT) nanocomposite

CS/Au/MWCNT nanocomposite containing different concentrations of Au as well as MWCNT *i.e.*, 5 wt% to 15 wt% and 5-FU@CS/Au/MWCNT were prepared following the same procedure reported earlier for the preparation of CS/Au and 5-FU@CS/Au nanocomposites^35^ except an additional step wherein, MWCNT was added to the solution of CS and allowed to swell for about 3 h before the addition of Au chloride. After this, the rest of the procedure was carried out as mentioned. The powder obtained was then characterized further.

## 3 Results and discussion

### 3.1 Structural investigation

#### 3.1.1 X-ray diffraction

**Fig. 1:**
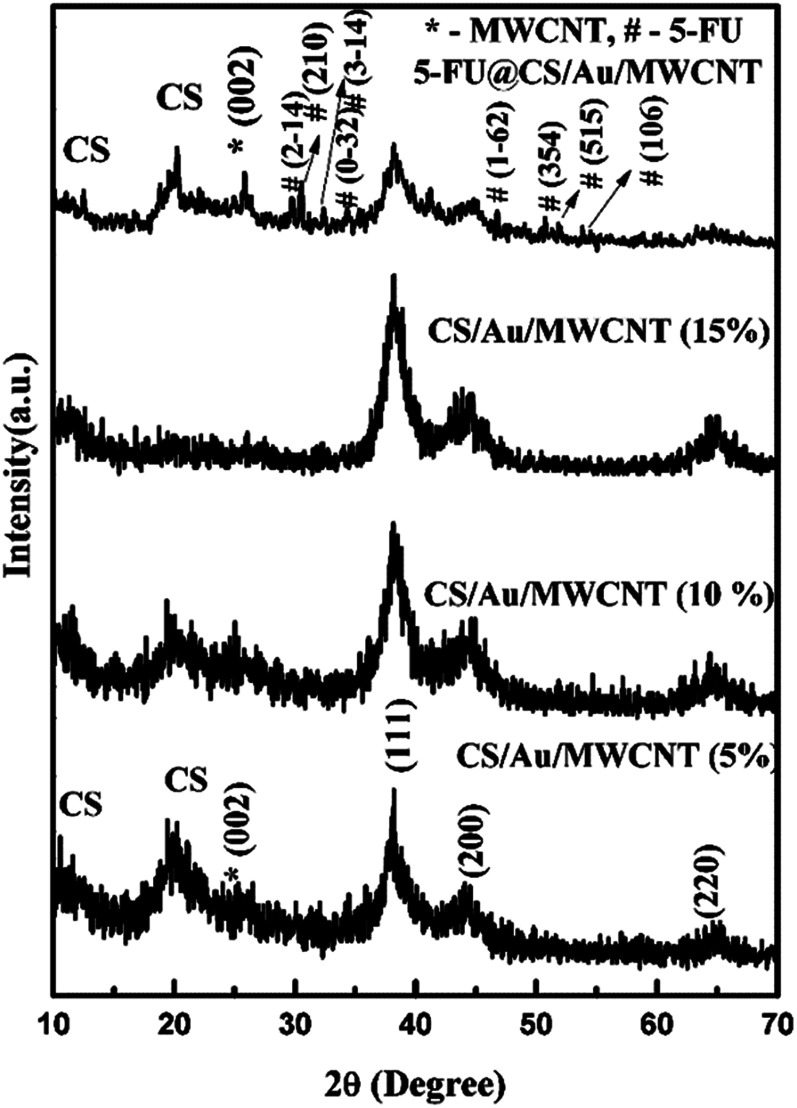
XRD pattern of CS/Au/MWCNT nanocomposite containing various percentages of Au, MWCNT and 5-FU encapsulated nanocomposite.

The XRD pattern of CS/Au/MWCNT and 5-FU@CS/Au/MWCNT are shown in Fig. 1. The XRD patterns of CS/Au/MWCNT, containing different concentrations of Au as well as MWCNT consists of 6 peaks, namely 2 peaks pertaining to CS at 2*θ* ∼11.8° and 21.0°, a single peak corresponding to the (002) reflection of graphite from MWCNT and 3 peaks of face centered cubic Au at 2*θ* = 38.1°, 44.4° and 64.5°. The peaks of Au are in good agreement with the JCPDS card no. 04-0784 and also with the already existing literature.^36,37^ The peak of MWCNT matches well with the literature reports.^38^ The average crystallite size of Au nanoparticles as calculated using Scherrer's formula is ∼5 nm. Appearance of peaks corresponding to all the three compounds evidences the formation of composite. In the case of 5-FU@CS/Au/MWCNT, peaks corresponding to 5-FU are observed in addition to the peaks of other moieties, affirming the encapsulation of 5-FU to the nanocomposite. The peaks of 5-FU concur well with the JCPDS card no. 39-1860.

#### 3.1.2 SAED analysis

**Fig. 2:**
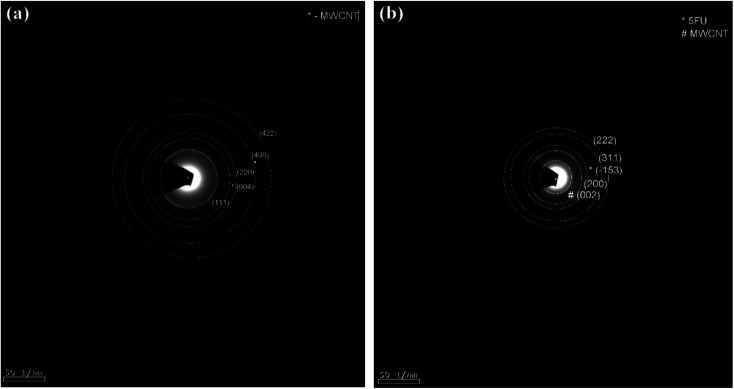
SAED pattern of (a) CS/Au/MWCNT nanocomposite and (b) 5-FU@CS/Au/MWCNTnanocomposite.

The SAED pattern of CS/Au/MWCNT and 5-FU encapsulated nanocomposite are shown in Fig. 2(a) and (b) respectively. The pattern of CS/Au/MWCNT shows the presence of (111), (220), (400) and (422) reflections of face centered cubic Au along with (004) reflection of graphite from MWCNT. The other reflections of Au are feeble so have not been indexed. The pattern obtained for the drug loaded nanocomposite shows the reflections from Au [(200), (311), (222)], MWCNT [(002)] as well as 5-FU [(−153)] confirming the encapsulation of 5-FU into the nanocomposite system. The results obtained here substantiate the XRD results, confirming the face centered cubic nature of Au and the formation of nanocomposite. The formation of nanocomposite is also evident from the XPS studies (presented in the ESI, refer Fig. S1†). The obtained results also affirm the encapsulation of 5-FU into the nanocomposite.

### 3.2 Particle size and zeta potential

**Fig. 3:**
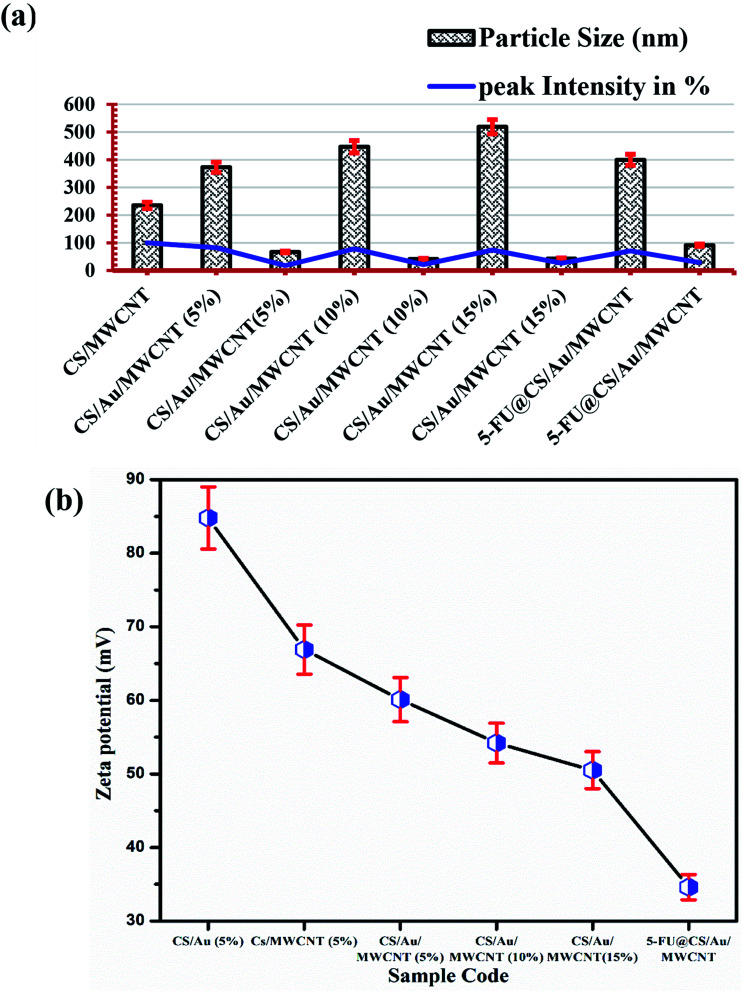
(a) Particle size measurements and (b) zeta potential of the prepared samples.

The particle size of the prepared samples along with the peak intensity has been presented in Fig. 3(a). Two particle size distributions are observed for the case of CS/Au/MWCNT composites. One at ∼400 nm and the other at ∼55 nm, which correspond to CS and/or MWCNT and Au nanoparticles respectively. The obtained particle sizes clearly indicate the successful synthesis of a nanocomposite system for the delivery of 5-FU. It is also observed that there is an increase in the size of the Au nanoparticles on encapsulation of 5-FU. This might be due to the binding of one 5-FU to more than one Au nanoparticle.

The zeta potential values obtained for all the samples is plotted in Fig. 3(b). It is observed that the zeta potential value of CS/Au as well as CS/MWCNT are highly positive, whereas there is a decrease in zeta potential on increasing the concentration of Au as well as MWCNT. This is attributed to the negative zeta potential observed for both Au^39^ and MWCNT^40,41^ as reported previously. Similarly, the zeta potential is seen to further decrease on the addition of 5-FU, having a negative zeta potential.^42^ The zeta potential of all the prepared systems still remains high indicating the high stability of the prepared nanocomposites.

### 3.3 Spectroscopic characterization

#### 3.3.1 UV-Vis studies

**Fig. 4:**
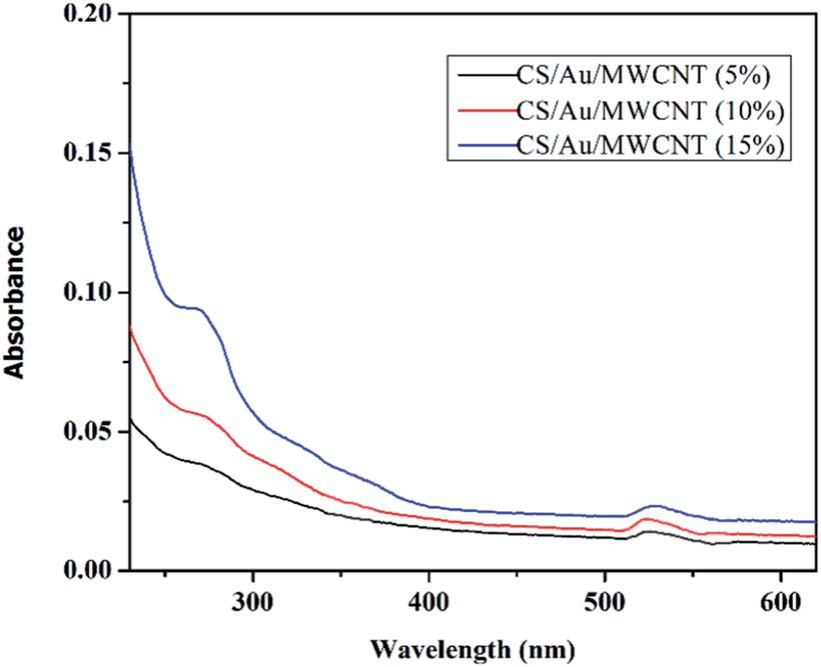
UV-Vis spectra of CS/Au/MWCNT nanocomposite containing various percentages of Au and MWCNT.

The UV-Vis spectra of CS/Au/MWCNT nanocomposite containing different weight percentage of Au (5–15 wt%) as well as MWCNT (5–15 wt%) are shown in Fig. 4. The results clearly show the presence of two absorption peaks one in the wavelength range 540–545 nm and the other at ∼265 nm. The peak in the range of 540–545 nm is attributed to the surface plasmon excitation of small spherical Au nanoparticles. An increase in the absorbance is observed on increasing the concentration of gold chloride during synthesis, thus implying an increase in the amount of Au binding to CS. A similar behavior has been reported previously.^43^ Although an increase in absorbance is observed, the peaks are damped in nature, indicating the small particle size. In small particles, the mean free path of the electrons is reduced which eventually leads to the peak dampening. The second peak at ∼265 nm corresponds to the absorbance from MWCNT.^44^ In a manner similar to the peaks of Au, the intensity of MWCNT peaks are also seen to increase on increasing the initial amount of MWCNT taken.

#### 3.3.2 FTIR studies

**Fig. 5:**
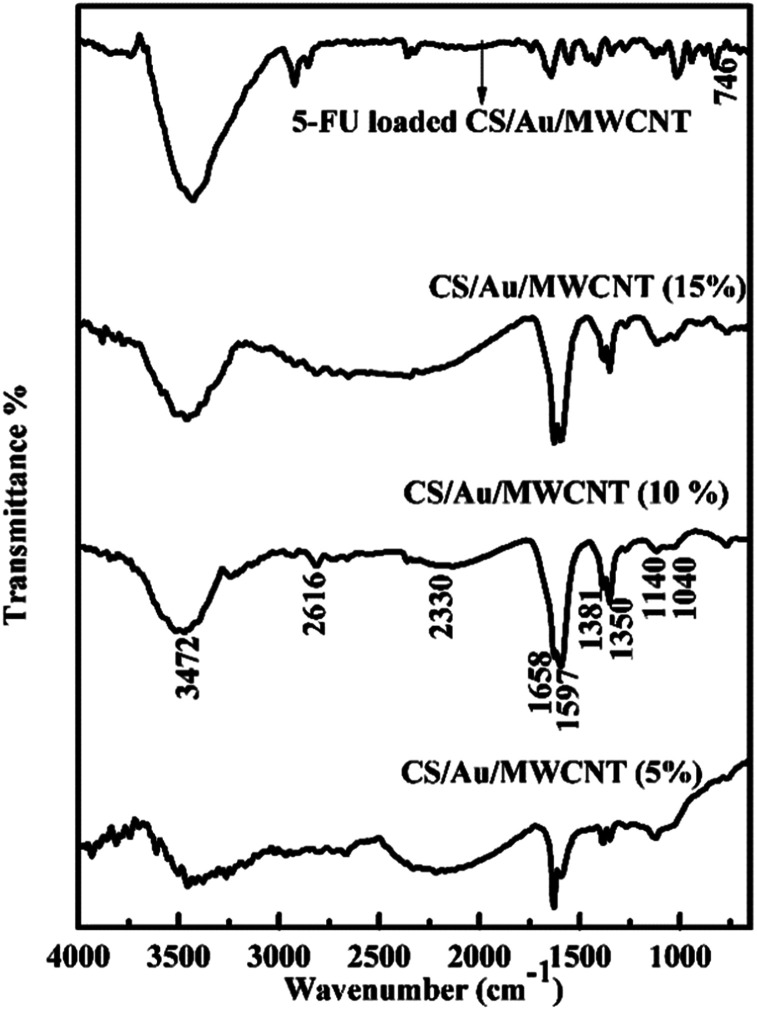
FTIR spectrum of CS/Au nanocomposite containing various percentages of Au, MWCNT and 5-FU encapsulated nanocomposite.

The FTIR spectrum of CS/Au/MWCNT nanocomposite containing different weight percentage of Au (5–15 wt%) as well as MWCNT (5–15 wt%) and that of 5-FU@CS/AU/MWCNT are shown in Fig. 5. All the spectra exhibit a NH_2_ twisting peak at ∼898 cm^−1^, C–O stretching at ∼1070 cm^−1^, C–O–C stretching at ∼1260 cm^−1^, C–N stretching at ∼1318 cm^−1^, C–H bending at 1382 cm^−1^, CH_2_ bending at ∼1416 cm^−1^, a peak of NH_3_^+^ at ∼1568 cm^−1^, C=O stretching and N–H bending at ∼1655 cm^−1^, C–H stretching at ∼2390 cm^−1^and N–H, O–H stretching at ∼3400 cm^−1^. These peaks correspond well to the peaks of pure CS except for minor differences that establish the formation of the nanocomposite systems,^45,46^ as has been previously reported by us for the case of CS/Au nanocomposite.^35^ The splitting of the NH_3_^+^ and NH_2_ peak increases on increasing the amount of MWCNT and/or Au in the nanocomposite. This is because of the neutralization of the protonated amine group. The values reported for the samples match closely to the previous reports.^47,48^ Change in intensity and shifting of the OH peak observed here, show the binding of MWCNT and/or Au to OH group of CS. The splitting of NH_2_ and NH_3_^+^ peaks is also noted, indicating the binding of MWCNT as well as Au to the NH_2_ groups of CS. The FTIR spectrum of 5-FU encapsulated nanocomposite shows the presence of a peak at ∼740 cm^−1^ corresponding to the C–H out of plane vibration of CF=CH in addition to the peaks observed for the nanocomposite as noticed in the case of CS/Au nanocomposite. This ascertains the encapsulation of 5-FU to the nanocomposite systems. Similar reports are also available in the literature.^49^

### 3.4 Morphology studies and elemental mapping

**Fig. 6:**
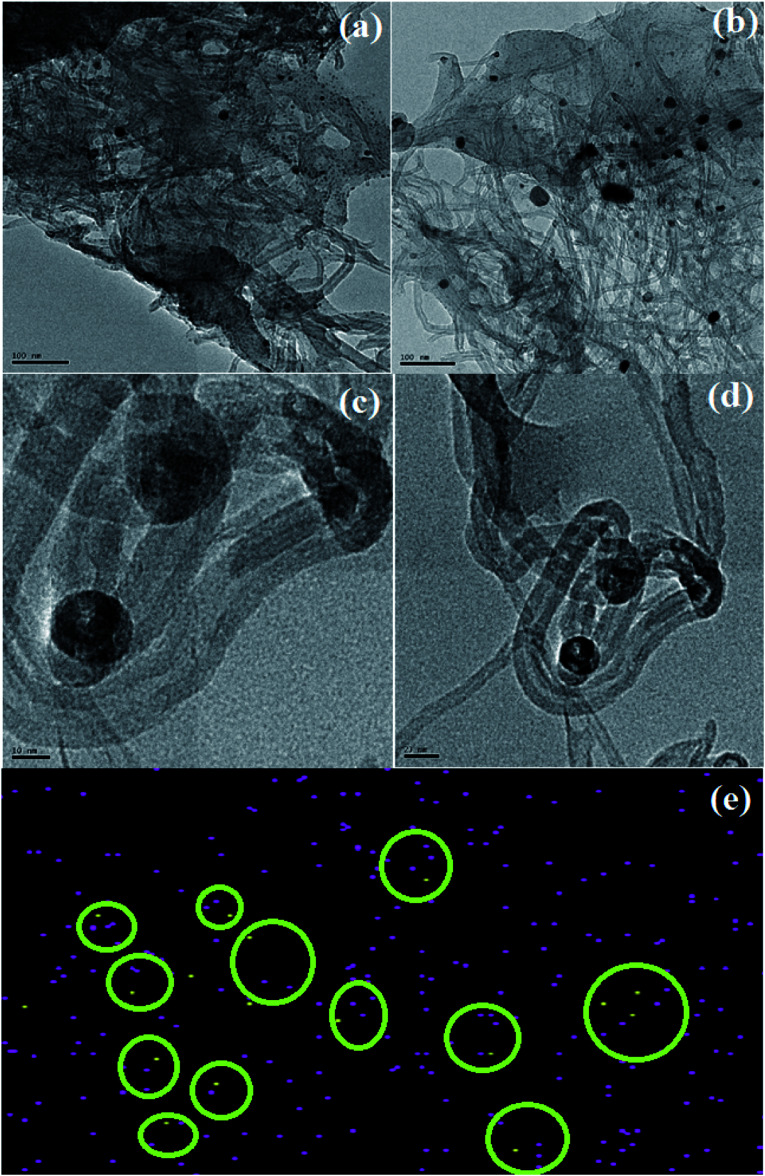
HRTEM image of (a) & (b) CS/Au/MWCNT nanocomposite showing the polymer matrix structure, (c) & (d) 5-FU@CS/Au/MWCNT nanocomposite showing the agglomeration of nanoparticles and increase in particle size and (e) elemental mapping of 5-FU@CS/Au/MWCNT showing Au and 5-FU.

The HRTEM images of CS/Au/MWCNT are shown in Fig. 6(a) and (b) and that of 5-FU@CS/Au/MWCNT are shown in Fig. 6(c) and (d). The formation of a polymer matrix type nanocomposite is evident from the images for the nanocomposite. The images clearly show MWCNT to be embedded in CS and the Au nanoparticles to be decorated on MWCNT as well on CS. A similar morphology wherein CS forms the matrix phase and the Au nanoparticles form the filler phase has been reported for the case of CS/Au nanocomposite. The particle size of Au nanoparticles as measured from the obtained images is ∼5 nm which is in good agreement with the XRD results and also supports the UV-Vis results obtained for the nanocomposite system. An increase in the particle size of Au nanoparticles to about 11 nm after the encapsulation of 5-FU is observed from the HRTEM images. Agglomeration of nanoparticles is also observed which is an indication of the binding of one 5-FU to more than one CS capped Au nanoparticle thus, bringing them closer to one another. This is evident from the elemental mapping the nanocomposite shown in Fig. 6(e). The increase in the particle size of Au observed here is in good agreement to the previous report on CS/Au nanocomposite. Apart from the increase in the size of Au nanoparticles, entanglement of MWCNT is also observed.

### 3.5 Evaluation of 5-Fluorouracil encapsulation and loading

Encapsulation efficiency of 5-FU is calculated using the formula mentioned elsewhere.^35^ The amount of free 5-FU has been obtained following the procedure reported previously by us for the case of 5-FU@CS/Au nanocomposite. The encapsulation efficiency of 5-FU into the nanocomposite is found to be 98% and the loading efficiency is found to be 43%. Both the values are seen to higher when compared to those obtained for 5-FU@CS/Au nanocomposite thus, showing the significance of incorporation of MWCNT into the nanocomposite.

### 3.6 5-Fluorouracil release studies

**Fig. 7:**
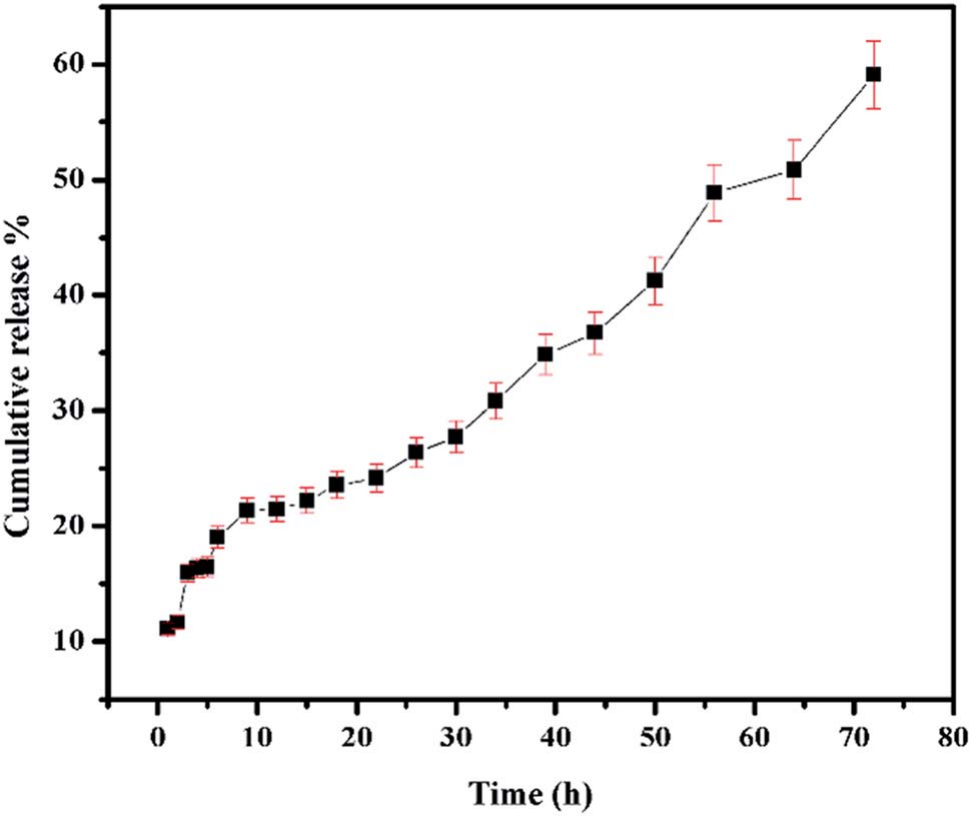
Drug release profile of 5-FU@CS/Au/MWCNT nanocomposite.

The conditions maintained when studying the release of 5-FU from CS/Au nanocomposite reported before have been maintained to study the release of 5-FU from CS/Au/MWCNT system as well.^35^ The release profile of 5-FU from CS/Au/MWCNT nanocomposite is shown in Fig. 7. Release of 5-FU from CS/Au/MWCNT shows a slow, sustained and prolonged release when compared to the release of 5-FU from CS/Au nanocomposite. The release profile is fitted to the various kinetics. According to the correlation values obtained after fitting the data into the various models, the data in the first region (region 1 : 1 to 6 h) of the release profile fitted well to the Higuchi kinetics, second region (region 2 : 12 to 30 h) adhered to Hixson–Crowell kinetics, third region (region 3 : 34 to 50 h) to zero order kinetics and the fourth region (region 4 : 56 to 72 h) to first order kinetics. A comparative study of the release profiles of 5-FU from CS/Au/MWCNT and CS/Au shows the release of about 99% of the drug from CS/Au nanocomposite in 72 h whereas only a 59% release is observed from CS/Au/MWCNT thus, confirming the effect of incorporation of MWCNT into the nanocomposite leading to the prolonged release time.

### 3.7 *In vitro* cytotoxicity analysis

**Fig. 8:**
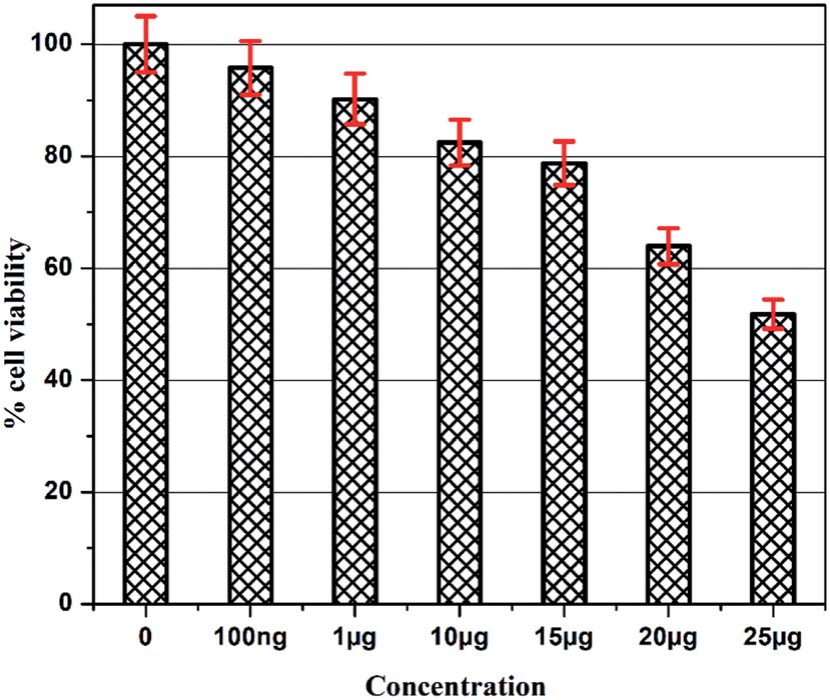
Cytotoxicity analysis of 5-FU@CS/Au/MWCNT nanocomposite towards MCF-7 cells.

**Fig. 9:**
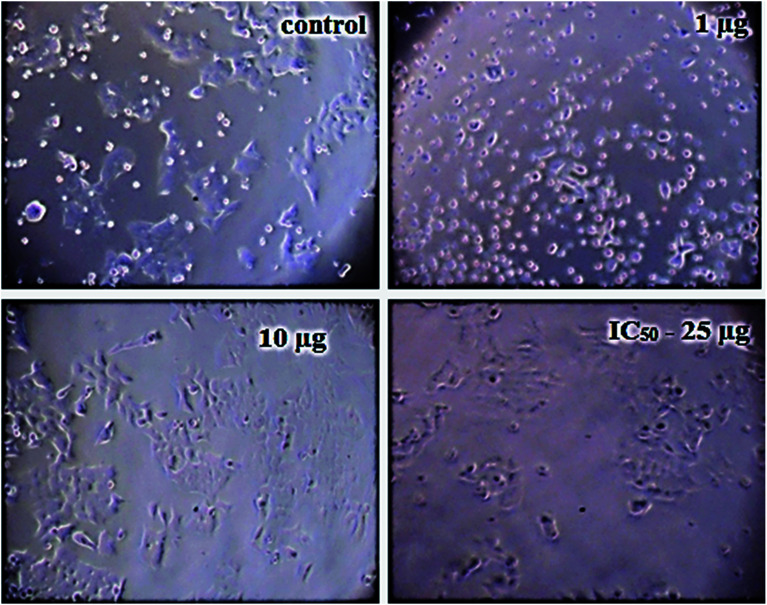
Images showing the cell viability of MCF-7 on addition of different concentrations of 5-FU@CS/Au/MWCNT nanocomposite.

The details of the cell lines and cell culture, the cytotoxicity assay procedure and the procedure followed for the calculation of % cell viability are similar to those reported previously.^35^ Viable cells were determined by measuring the absorbance at 540 nm using UV-Vis spectrophotometer. Measurements were performed and the concentration required for a 50% inhibition of viability (IC_50_) was determined graphically. Fig. 8 shows the result of cytotoxicity measurement obtained for the MCF-7 cell lines, performed 24 h after the addition of 5-FU@CS/Au/MWCNT nanocomposite. Fig. 9 shows the images obtained after the addition of different concentrations of 5-FU encapsulated nanocomposite. CS/Au/MWCNT nanocomposite exhibits a concentration dependent loss of viability. The estimated half maximal inhibitory concentration (IC_50_) value for the nanocomposite system is found to be 25 μg ml^−1^. This nanocomposite system is seen to be more cytotoxic at lower concentrations when compared to the CS/Au nanocomposite system. CS/Au/MWCNT is seen to have a better antiproliferative effect on MCF-7 due to the fact that MWCNT can perforate cellular membrane and pass into the cellular components without causing apparent cell damage.

## 4 Conclusion

CS/Au/MWCNT nanocomposite with and without 5-FU encapsulation were successfully prepared by the chemical route. The semi-crystalline nature of CS, face centered cubic structure of Au are affirmed from the XRD patterns. The presence of MWCNT in the nanocomposite and the triclinic structure of 5-FU in the drug loaded nanocomposite is confirmed using the XRD pattern. Binding of the moieties to the OH and NH_2_ groups of CS is evident from the FTIR spectra and XPS spectrum. The formation of polymer matrix nanocomposite and an increase in the particle size of Au and the entanglement of carbon nanotubes on the encapsulation of 5-FU are evident from the HRTEM images. The encapsulation and loading efficiency were found to be 98% and 43% for 5-FU loaded CS/Au/MWCNT nanocomposite. These are found to be higher than the values reported for CS/Au as well as CS/Ag/MWCNT. A comparatively prolonged and sustained release and a better cytotoxicity was exhibited by 5-FU encapsulated CS/Au/MWCNT nanocomposite when compared to the previously reported system containing CS and Au. The CS/Ag/MWCNT system, proved the effectiveness of this ternary system for delivering 5-FU and inhibiting MCF-7.

## Author contribution statement

E. A. K. N. Conceptualization, planning of the investigation, data collection and analysis. Preparation of the manuscript with the suggestion from the other authors. S. D. and J. S. helped in the synthesis and characterization. C. A. M. helped in synthesis and the biological characterization. V. N. and S. N. K. critically revised the article. N.S helped in performing the UV-VIS characterization. AS Planning, administration of the work and critical review of the manuscript. All the authors have read and approved the final version of this manuscript.

## Conflicts of interest

There are no conflicts to declare.

## Supplementary Material

RA-011-D0RA08537E-s001
