# Supplementary material for: CS/Au/MWCNT nanohybrid as an efficient carrier for the sustained release of 5-FU and a study of its cytotoxicity on MCF-7
Source: RSC Adv. 2021 Jan 22;11(8):4584–92. doi: 10.1039/d0ra08537e (PMC8694463; doi:10.1039/d0ra08537e)
Supplement: RA-011-D0RA08537E-s001 [file RA-011-D0RA08537E-s001.pdf]

Electronic Supplementary Material (ESI) for RSC Advances.
This journal is © The Royal Society of Chemistry 2021

CS/Au/MWCNT nanohybrid as an efficient carrier for the sustained release of 5-FU and a study of its cytotoxicity on MCF-7

E A K Nivethaa [a,d], S Dhanavel [b,d], V Narayanan [c], S Narayana Kalkura[e], J Siva Sankari [a], N Sivanandham[a] and A Stephen[d,*]

[a]*Department of Physics. Anna University, Guindy, Chennai-25, India*
[b]*Materials Chemistry & Metal Fuel Cycle Group, Indira Gandhi Centre for Atomic Research, Kalpakkam, India*
[c]*Department of Inorganic Chemistry, University of Madras, Guindy Campus, Chennai 600 025, India.*
[d] *Department of Nuclear Physics, University of Madras, Guindy Campus, Chennai-25, India*
[e]*Crystal Growth Centre, Anna University, Guindy, Chennai-25, India.*

**E-mail: stephen_arum@hotmail.com , stephen@unom.ac.in*
Phone: 044-22202802, Fax. 044-22351269

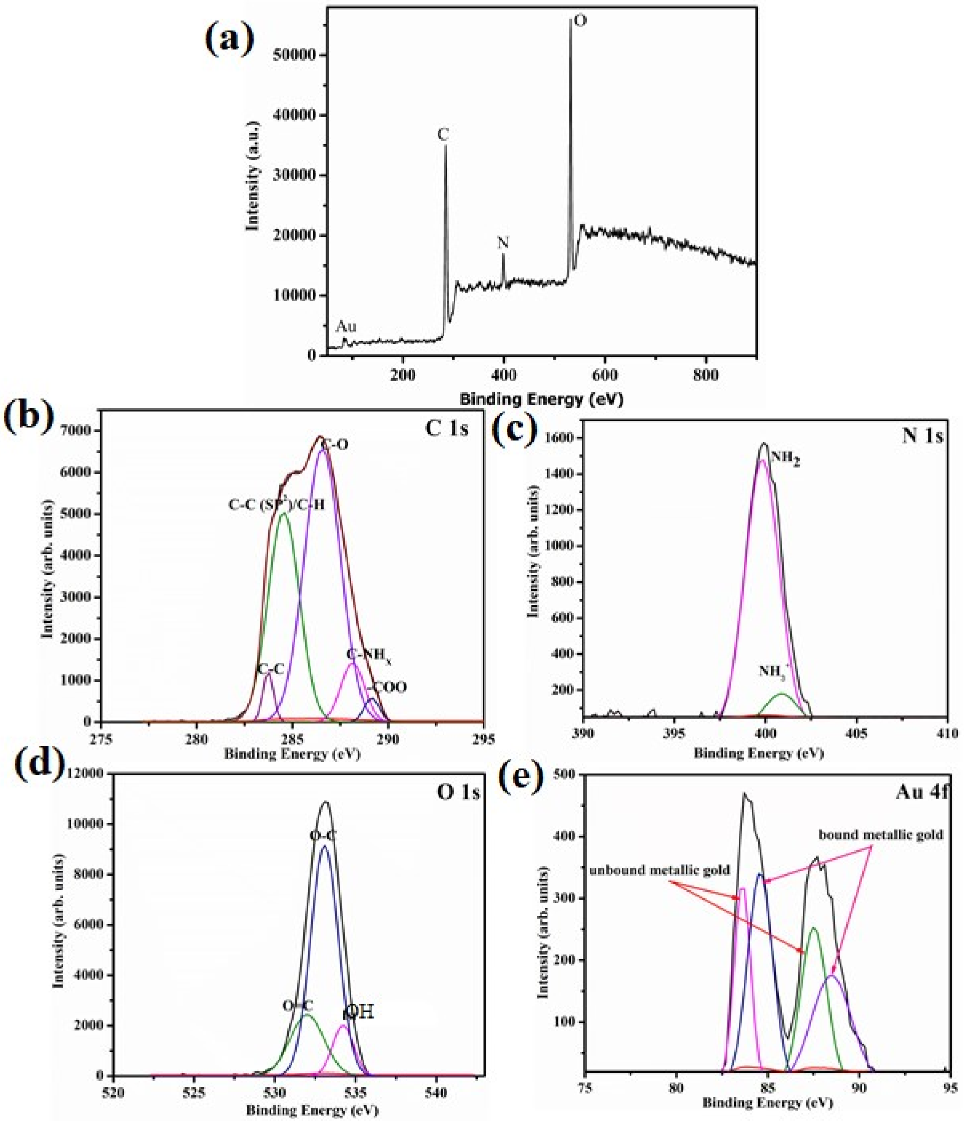

Figure S1. XPS spectrum (a) survey scan, (b) broad scan of C, (c) broad scan of N, (d) broad scan of O and (e) broad scan of Au.

The XPS spectrum of CS/Au/MWCNT nanocomposite is presented in figure S1. The survey spectrum shows the presence of C, N, O and Au and the absence of impurities in the prepared samples. Charge correction was performed taking carbon as the reference. The C 1s spectra is deconvoluted into 5 peaks namely, C-C at 284.0 eV, C-C (SP^2)/C-H peak at 284.6 eV, C-O peak at 286.5 eV, C-NH_X peak at 288.1 eV and -COO peak at 289.1 eV. The fitting of the N 1s spectrum yielded 2 peaks having a binding energy of 399.7 and 400.8 eV, which correspond to NH_2 and NH_3^+, respectively. NH_3^+ occurs due to the protonation of amine groups of chitosan. A shift of the NH_2 and NH_3^+ peaks by 0.7 eV and 0.8 eV, respectively when compared to pure chitosan, elucidates the binding of Au and/or MWCNT to the amine group of chitosan. The O 1s spectrum consists of O=C, O-C and O-H peaks at 531.9, 533.0 and 534.2 eV, respectively. A shift in the OH peak by 0.9 eV indicates the binding of Au and or MWCNT to it. The Au 4f spectrum shows the presence of both $4f_{7/2}$ (83.7 eV) and $4f_{5/2}$ (87.3 eV) peaks separated by 3.6 eV which is the characteristic of metallic gold. Apart from this the gold spectrum is also deconvoluted into two more peaks (84.7 eV and 88.3 eV), separated by a binding energy of 3.6 eV. These peaks also belong to the Au^0 state of gold, but are shifted from the original binding energy values of metallic gold by 0.9 eV due to the binding of gold nanoparticles to chitosan and also due to the particle size effect.
